# Enhancing learning outcomes through post-discussion homework: the role of collaboration and task type

**DOI:** 10.3389/fpsyg.2025.1666213

**Published:** 2026-01-05

**Authors:** Xuejiao Cheng, Ruizhu Yuan, Qihao Zhang, Chengxi Zhai

**Affiliations:** 1 School of Discipline Inspection and Supervision, Sichuan Normal University, Chengdu, China; 2 Discipline Inspection and Supervision Research Center, Chengdu, China; 3 Sichuan Key Laboratory of Psychology and Behavior of Discipline Inspection and Supervision, Chengdu, China; 4 Department of Applied Psychology, Hubei University of Medicine, Shiyan, China

**Keywords:** collaborative learning, homework design, inclusive pedagogy, instructional design, task type

## Abstract

This study investigates how post-discussion homework can be designed to sustain the inclusive, collaborative dynamics of seminar teaching. Through a 2 × 2 quasi-experimental design with 36 graduate students, we examined how social architecture (collaborative vs. individual homework) and task type (concept-oriented vs. case-oriented assignments) jointly relate to learning outcomes. Learning was assessed through rubric-based assignment performance, students’ perceived learning ratings, and brief written open-ended interviews about collaborative homework. Across assignments, collaborative homework was associated with higher performance than individual homework, and this collaborative advantage was especially pronounced for the concept-oriented task, which required students to reorganize and articulate theoretical ideas, compared with case-oriented assignments that emphasized applying established concepts to specific organizational cases. These findings suggest that while a collaborative social architecture is broadly beneficial, its potential for fostering deep learning and inclusive engagement appears to be strongest when it is paired with conceptually demanding work. The study offers an evidence-based framework for designing post-discussion homework as an integral phase of a continuous, collaborative learning cycle rather than as an isolated individual task.

## 1 Introduction

Collaborative and discussion-based approaches to teaching have become a prominent feature of contemporary higher education because they can promote academic achievement and more inclusive participation (Klang et al., 2020; Slavin, 2014). In seminar-style courses in particular, structured opportunities to think with peers help students articulate ideas and encounter alternative viewpoints. Through such interactions, they can co-construct understandings that they might not reach on their own (Howe and Abedin, 2013; Wells and Arauz, 2006). Meta-analytic and review work on cooperative learning in universities generally shows that, on average, students in small-group or cooperative structures perform at least as well as, and often better than, students in purely individual formats and that they tend to report more positive attitudes toward learning and stronger engagement (Kyndt et al., 2013; Springer et al., 1999). Within this literature, Social Interdependence Theory provides a central lens for understanding how patterns of interdependence and accountability shape the quality and outcomes of collaboration (Deutsch, 1949; Johnson and Johnson, 2002, 2005, 2009a).

Much of this evidence, however, comes from research on activities that occur during scheduled class time—such as small-group discussions, cooperative problem-solving tasks, and other forms of structured in-class interaction (Gillies, 2016; Slavin, 2014; Smith et al., 2005; Xu et al., 2023). In contrast, relatively little attention has been paid to how collaborative learning is organized in the homework phase, even though homework occupies a substantial proportion of students’ study time and often determines how ideas from class are consolidated into written products. Research on homework has tended to emphasize the amount of homework, time spent, or the relationship between homework and achievement (Cooper et al., 2006; Esen et al., 2023), with far less focus on how the design of homework might extend or dilute the benefits of in-class collaboration. This gap is particularly salient in discussion-based courses, where a great deal of conceptual work already takes place through peer interaction during class, yet post-discussion homework is often treated as an individual add-on rather than as a deliberate continuation of collaborative learning.

Designing post-discussion homework in a more systematic way requires attention to at least two core dimensions. The first is the social architecture of the assignment—that is, whether students are expected to work individually or collaboratively and how responsibility for the final product is organized. In many university courses, homework is an individual task, and each student submits their own work for grading. In other courses, or for particular assignments, students are asked to work together and submit a single shared product on behalf of the group. From a Social Interdependence perspective, these arrangements differ in the extent to which they create opportunities for positive interdependence and shared responsibility, as well as promotive interaction in which students exchange resources, provide feedback, and challenge one another’s reasoning (Deutsch, 1949; Johnson and Johnson, 2002, 2005, 2009a). When homework is structured around a shared written product and expectations for participation are clear, students have reasons to coordinate their efforts, explain ideas to one another, and monitor each other’s contributions beyond the classroom session (Cohen, 1994; Kyndt et al., 2013; Premo et al., 2018; Scager et al., 2016). At the same time, collaborative homework can introduce coordination demands and concerns about free riding or fairness, and without adequate support these costs may offset potential gains (Barron, 2003; Omodan, 2020; Radkowitsch et al., 2020; Yang, 2023). Empirically, evidence is still limited on when collaborative homework yields better learning than individual homework in authentic university settings and under what conditions it might instead generate frustration or “collaborative fatigue.”

A second design dimension concerns the type of task and its associated cognitive demands. Work on task structure and conceptual change often distinguishes between relatively well-structured, application-oriented tasks and more ill-structured tasks that require students to construct and reorganize knowledge (Jonassen, 2000; Reed, 2016). In higher education, these broad categories are frequently reflected in two familiar forms of assignment. Case-oriented tasks present a concrete scenario and ask students to use course concepts to diagnose problems and propose responses—for example, analyzing an organization’s difficulties and recommending interventions in light of guiding questions. Such tasks tend to channel students toward applying an existing conceptual framework to a particular situation and often invite more convergent thinking (Crogman and Trebeau Crogman, 2018; Reed, 2016). Concept-oriented tasks, in contrast, ask students to work directly with theoretical ideas by explicating core concepts, comparing alternative perspectives, and organizing them into an integrated account, often resembling “wicked” or ill-structured problems that demand higher levels of abstraction and synthesis (Jonassen, 2000; Reed, 2016; Veltman et al., 2021).

Socio-Cognitive Conflict Theory suggests that, in collaborative settings, these differences in task type matter for how and how much students learn together. When tasks are relatively well structured and application-oriented, group work may help distribute workload and provide mutual support, but interaction can be dominated by dividing sub-tasks and assembling partial answers. Because concept-oriented tasks are more open-ended and ill-structured, they are more likely to elicit discrepant interpretations and uncertainty. When students must explain, justify, and reconcile different views about a concept, they may experience productive “epistemic conflict,” which in turn prompts them to refine their reasoning and reorganize their conceptual representations (Butera et al., 2019; Johnson and Johnson, 2009b). From this standpoint, collaborative work on concept-oriented tasks may be especially conducive to deeper conceptual learning, whereas collaborative work on case-oriented tasks may primarily facilitate the application and refinement of already familiar frameworks (Ludvigsen et al., 2018; Malone and Michael, 2018; Rodriguez-Dono and Hernández-Fernández, 2021; Zhu et al., 2023).

Bringing these two dimensions together suggests that the effects of collaborative homework are unlikely to be uniform across tasks. Collaborative homework may be generally beneficial compared with individual homework because it can extend opportunities for explanation, feedback, and shared regulation beyond the classroom (Cohen, 1994; Kyndt et al., 2013; Premo et al., 2018; Richey et al., 2018; Springer et al., 1999). However, theoretical accounts also imply that collaborative advantages should be stronger when homework requires students to reorganize and articulate theoretical ideas in an open-ended way than when it primarily involves applying well-understood concepts to structured cases (Jonassen, 2000; Reed, 2016). This perspective complements work on team structure and task type showing that how tasks are designed can shape the benefits of collaboration (Stewart and Barrick, 2000). Despite this theoretical rationale, there is still relatively little empirical work that examines how the mode of homework completion (collaborative vs. individual) interacts with task type (concept-oriented vs. case-oriented assignments) in real courses, especially in graduate-level, discussion-based courses and in non-Western higher-education contexts.

A further issue concerns how learning from post-discussion homework is assessed. Much of the work on cooperative and collaborative learning relies on performance measures such as test scores or assignment grades. These outcomes are essential, but in the context of inclusive and student-centered pedagogy, students’ perceived learning—their own judgments of the extent to which particular activities help them understand and make sense of course content—is also a meaningful indicator. This view is consistent with longstanding work in higher education assessment treating students’ self-reported learning and course experience as important complements to achievement data (Richardson, 2005). Recent work in collaborative learning and distance-education settings has treated perceived learning as a relevant outcome alongside performance, emphasizing that how students experience learning activities can matter as much as how they perform on tests (Ahmed Abdel-Al Ibrahim et al., 2023; Saw and Mohamad, 2024). Perceived learning is not a substitute for achievement, but it can complement performance measures in at least two ways. First, it reflects whether students experience particular forms of homework as educationally worthwhile, which is especially relevant when collaborative homework requires additional time and coordination compared with individual work. Second, tasks that students perceive as valuable are more likely to be sustained over time and integrated into their broader learning strategies, as suggested by studies of collaborative engagement and perceived learning in university and online environments (Asad and Malik, 2024; Qureshi et al., 2023). Nevertheless, empirical studies that simultaneously consider performance and perceived learning in relation to collaborative versus individual post-discussion homework remain limited.

**Figure 1:**
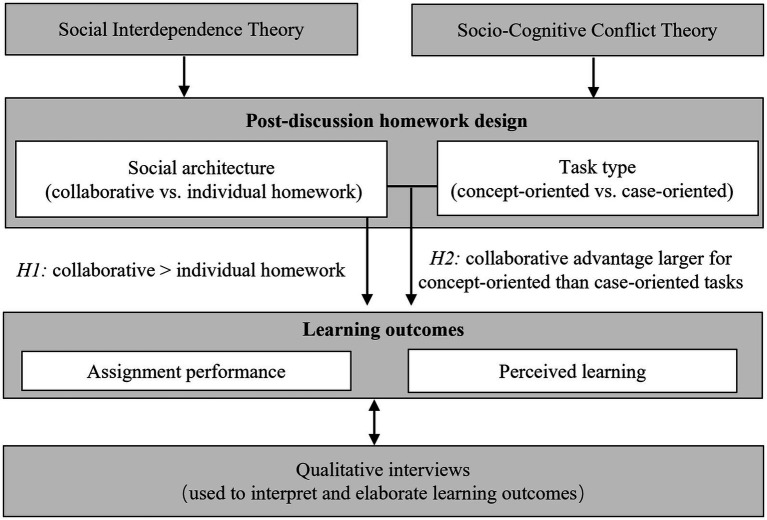
Conceptual model of post-discussion homework and learning outcomes.

Against this backdrop, the present study examines post-discussion homework in a discussion-based graduate seminar at a university in China. In this authentic course setting, students regularly engaged in structured in-class discussions and then completed written homework that built directly on those discussions. Post-discussion homework took two forms: one concept-oriented assignment that asked students to reorganize and articulate theoretical ideas in an ill-structured way, and two case-oriented assignments that asked them to apply those ideas to authentic organizational cases. Within the ecological constraints of a single intact cohort, we contrasted collaborative and individual modes of completing these assignments. We focused on two complementary outcomes—rubric-based assignment performance and students’ perceived learning—and supplemented these with brief written open-ended interviews about students’ experiences of collaborative homework. Figure 1 presents the conceptual model that guided the study, linking Social Interdependence Theory and Socio-Cognitive Conflict Theory to the design of post-discussion homework (social architecture and task type) and to learning outcomes (assignment performance and perceived learning), with qualitative interviews used to interpret and elaborate these outcomes (Butera et al., 2019; Johnson, 2003).

Guided by these perspectives, we formulated two hypotheses. Drawing on Social Interdependence Theory, we expected collaborative homework to confer advantages over individual homework across assignments. Specifically, students who completed homework collaboratively were expected to achieve higher assignment performance and report greater perceived learning than those who worked individually, because collaborative homework extends opportunities for promotive interaction, shared responsibility, and mutual regulation beyond the classroom session (H1). This expectation is consistent with foundational work on social interdependence and cooperative learning (Deutsch, 1949; Johnson and Johnson, 2009a).

Building on socio-cognitive conflict accounts of task demands, we further anticipated that the magnitude of this collaborative advantage would vary as a function of task type. In particular, collaboration was expected to be especially beneficial for the concept-oriented assignment, which required students to reorganize and articulate theoretical ideas in an ill-structured way, relative to case-oriented assignments that primarily involved applying established concepts to specific organizational scenarios (H2). This task-specific prediction follows from theoretical work emphasizing the role of epistemic conflict and elaboration in conceptually demanding collaborative tasks (Butera et al., 2019; Johnson and Johnson, 2009b).

## 2 Method

### 2.1 Participants and design

Participants were 36 graduate students in psychology (27 women, 9 men; age 21–26 years) enrolled in a seminar titled *Organizational Learning and Knowledge Management* at a public comprehensive university in China. The seminar combined lectures, structured discussions, and written assignments, and all study activities were embedded in the regular course. Students worked throughout the semester in intact project teams. All participants provided informed consent, and the study was approved by the university’s ethics committee.

The study employed a 2 × 2 quasi-experimental design with two factors: task type (concept-oriented vs. case-oriented) and social architecture (collaborative vs. individual homework). Task type varied across the assignments, whereas social architecture was manipulated between teams. Given the modest sample size and the intact-team structure, we focused on planned comparisons targeting our two hypotheses rather than fitting a full factorial ANOVA model.

At the beginning of the semester, students were randomly assigned to six project teams (g1–g6; six students each), which were maintained throughout the course. Teams were then allocated to two counterbalanced sequences to ensure that (a) all students experienced both collaborative and individual homework and (b) the order of collaborative versus individual work for the case assignments was balanced across teams. Table 1 summarizes the implementation schedule. The sample represents a single intact cohort, which limits generalizability but reflects the authentic instructional setting in which the design was implemented.

**Table 1:** Experimental design and counterbalancing scheme.

Task sequence	Assignment 1 (Week 5)	Assignment 2 (Week 11)	Assignment 3 (Week 18)
	Concept-oriented	Case-oriented	Case-oriented
Sequence A (*n* = 18; groups g1–g3)	Collaborative	Individual	Collaborative
Sequence B (*n* = 18; groups g4–g6)	Individual	Collaborative	Individual

### 2.2 Procedure and task materials

#### 2.2.1 Research procedure

At the start of the semester, students were randomly assigned to six project teams of equal size. The experimental manipulation concerned only the mode of homework completion; all other instructional activities (lectures, discussions, in-class exercises) followed the regular course plan.

Across the semester, students completed three major written assignments aligned with the course sequence: a concept-oriented assignment in Week 5 and two case-oriented assignments in Weeks 11 and 18. The counterbalanced sequences shown in Table 1 determined whether each assignment was completed collaboratively or individually. In Sequence A, the concept-oriented assignment and the second case assignment were completed collaboratively, whereas the first case assignment was completed individually. Sequence B followed the opposite pattern. Thus, every student completed one concept-oriented assignment and two case-oriented assignments, and each student experienced both collaborative and individual homework in a counterbalanced order.

For collaborative assignments, each team produced a single joint report outside of class; for individual assignments, students worked independently and submitted their own reports. Immediately after submitting each assignment—and before receiving grades or feedback—students rated their perceived learning for that assignment. For collaboratively completed assignments, the response sheet also included a brief written open-ended interview prompt, asking students to describe how they worked with their peers and what they felt they had learned. These written interview responses provided the qualitative material for the study.

#### 2.2.2 Task materials

##### 2.2.2.1 Concept-oriented assignment

The concept-oriented assignment required students to synthesize theoretical ideas from the early units of the course. Students articulated what constitutes a “learning organization,” integrated perspectives from assigned readings, and explained how these concepts relate to organizational change and knowledge management. The task was designed to elicit higher-order conceptual reasoning rather than application to a specific scenario; students were expected to construct a coherent conceptual account, compare and connect theoretical perspectives, and organize them into an integrated framework.

##### 2.2.2.2 Case-oriented assignments

The two case-oriented assignments required students to apply course concepts to authentic organizational cases. The first case examined innovation-management challenges in Legend Group, and the second focused on knowledge-management and knowledge-sharing issues within International Business Machines Corporation (IBM). For each case, students analyzed core organizational problems, interpreted them through theoretical frameworks discussed in class, and proposed evidence-informed interventions. The Legend Group case emphasized the tension between sustaining innovation and maintaining internal coordination, whereas the IBM case highlighted barriers to knowledge flow across units. Before the semester began, the course instructor and a senior faculty member in organizational psychology jointly reviewed the cases to ensure that they were comparable in length, complexity, and cognitive demand. Because the two cases addressed different organizations at different points in the semester, they were treated as separate learning episodes rather than repeated measures of the same task.

#### 2.2.3 Measures

##### 2.2.3.1 Assignment performance

Performance on each assignment was graded on a 0–100 scale using a set of analytic criteria and scoring guidelines developed by the course instructor (a senior professor in organizational psychology) in consultation with the first author, to align instructional expectations with the aims of the study. A common set of core criteria—conceptual accuracy, depth of analysis, appropriate use of theory, quality of argumentation, and evidential justification—was applied across all assignments. Task-specific guidance translated these general criteria into concrete indicators for the concept-oriented and case-oriented tasks while maintaining comparable standards of rigor.

Before scoring the study assignments, the course instructor and a trained research assistant jointly reviewed exemplar reports from a prior cohort to calibrate their use of the analytic criteria and ensure a shared understanding of performance levels. The two raters then independently scored all 108 assignments using the same rubric. Raters were blind to whether a given report had been produced collaboratively or individually and to the sequence to which the team belonged. Inter-rater reliability for the two sets of scores across the full dataset was acceptable to good, *ICC* (3.1) = 0.72, 95% CI [0.61, 0.80], *p* < 0.001; the corresponding average-measures coefficient was *ICC* (3.2) = 0.83, 95% CI [0.76, 0.89]. After reliability had been established, the raters met to discuss and resolve any remaining discrepancies, and the resulting consensus scores were used in all analyses. For collaborative assignments, the final score assigned to the team report was applied to all team members.

##### 2.2.3.2 Perceived learning

Immediately after each assignment submission, students rated the extent to which the assignment helped them understand and make sense of course content on a 5-point scale (1 = not at all, 5 = very much). This single item provided a concise indicator of perceived learning at the assignment level in an authentic instructional context and minimized additional demands on class time. Ratings were collected from all students for all assignments. In the analyses, one rating per student was used for the concept-oriented assignment, and one rating per assignment submission was used for the two case-oriented assignments, in parallel with the performance scores.

##### 2.2.3.3 Qualitative interviews

For each collaboratively completed assignment, students responded to a brief written open-ended question embedded in the worksheet. The prompt asked them to describe how they worked with their peers and what they felt they had learned from the assignment. These written responses were part of regular coursework and constituted the qualitative data for the present study. In total, 54 written responses were collected (two collaborative assignments in Sequence A and one in Sequence B). All responses were written in Chinese; analysis was conducted in the original language, and illustrative excerpts were subsequently translated into English and checked by a bilingual colleague.

### 2.3 Data analysis

All quantitative analyses were conducted in IBM SPSS Statistics 29. For performance scores and perceived learning ratings, we first inspected descriptive statistics and normal Q–Q plots. No missing data were observed, and the distributions approximated normality. Levene’s test was used in all comparisons to decide whether to apply the standard or Welch-corrected *t*-test.

To examine the main effect of social architecture (H1), independent-samples *t*-tests compared collaborative and individual conditions separately for the concept-oriented assignment and for the case-oriented assignments. For the concept-oriented assignment, the unit of analysis was the student (18 collaborative vs. 18 individual scores). For the case-oriented assignments, each assignment submission was treated as a performance episode, yielding 36 collaborative and 36 individual performances across the two case tasks. This episode-level approach is common in small-scale classroom research because it preserves the ecological structure of repeated coursework and increases statistical power in modest samples. At the same time, we note that episodes are nested within students and groups, so the assumption of strict independence is only approximately met in this design.

To explore task-specific collaborative differences (H2) within the collaborative social architecture, we treated task type as a design-based proxy for cognitive demand: in the course, the concept-oriented assignment was constructed as an ill-structured, integrative task, whereas the two case-oriented assignments emphasized the application of established concepts to structured organizational scenarios (see Section 2.2.2). Within the collaborative condition, we compared performance on the concept-oriented assignment (18 collaborative episodes) with performance on the two collaborative case assignments (36 episodes: 18 from Sequence A and 18 from Sequence B) using an independent-samples *t*-test, applying the standard or Welch-corrected statistic based on Levene’s test. As a robustness check, we also aggregated each student’s collaborative case scores to the student level and repeated the comparison; the pattern of results was similar, and we report the episode-level analyses for consistency with the implementation of the tasks while acknowledging the nested structure as a limitation below.

For all *t*-tests, effect sizes were reported as Cohen’s *d* based on the pooled standard deviation of the two groups, providing a standardized indicator of the magnitude of between-condition differences (Cohen, 1988). Perceived learning ratings were analyzed using comparisons parallel to those for performance scores, with one rating per student for the concept-oriented assignment and one rating per assignment submission for the two case-oriented assignments. Given the modest sample size and quasi-experimental nature of the study, emphasis is placed on the convergence of patterns across measures and the size of effects rather than on fine-grained *p*-value thresholds.

Qualitative interview data were analyzed using reflexive thematic analysis in line with Braun and Clarke’s approach (Braun and Clarke, 2006, 2019). Two researchers independently read all written responses, generated initial data-driven codes, and then met to compare and refine a common set of codes. Codes were grouped into broader themes informed by Social Interdependence Theory and Socio-Cognitive Conflict Theory. Discrepancies were resolved through discussion until consensus was reached. For reporting, representative excerpts were translated from Chinese into English by the first author and checked by a bilingual colleague to preserve the meaning of the original text. Anonymized datasets are available from the corresponding author upon reasonable request.

## 3 Results

Table 2 presents the descriptive statistics for assignment performance and perceived learning as a function of task type (concept-oriented vs. case-oriented) and social architecture (collaborative vs. individual). Across both measures, collaborative conditions yielded higher mean values than individual conditions. The performance advantage of collaboration was evident for both concept-oriented and case-oriented assignments, and perceived learning ratings showed a broadly similar pattern.

**Table 2:** Descriptive statistics for assignment performance and perceived learning.

Measure	Task type	Social architecture	*N*	*M*	*SD*
Assignment performance	Concept-oriented	Collaborative	18	92.33	2.07
		Individual	18	85.86	4.01
	Case-oriented	Collaborative	36	89.33	3.75
		Individual	36	85.07	3.33
Perceived learning	Concept-oriented	Collaborative	18	4.39	0.50
		Individual	18	3.89	1.02
	Case-oriented	Collaborative	36	4.25	0.65
		Individual	36	3.81	1.06

Assignment scores range from 0 to 100. Perceived learning ratings were made on a 5-point Likert-type scale.

### 3.1 Main effect of social architecture (H1)

Our first hypothesis (H1) predicted that a collaborative social architecture would be associated with higher performance and perceived learning. Independent-samples *t*-tests were conducted separately for the concept-oriented and case-oriented tasks. The results supported this prediction for both task types.

For the concept-oriented task, students working collaboratively achieved higher scores (*M* = 92.33, *SD* = 2.07, *n* = 18) than those working individually (*M* = 85.86, *SD* = 4.01, *n* = 18). Levene’s test indicated unequal variances, so Welch’s corrected test was used, *t*(25.49) = 6.09, *p* < 0.001. The effect size was large (Cohen’s *d* = 2.03), indicating a pronounced performance advantage for collaborative homework on this conceptually demanding task.

For the case-oriented tasks, when performance episodes across the two case tasks were combined, the collaborative condition (*M* = 89.33, *SD* = 3.75, *n* = 36) also scored higher than the individual condition (*M* = 85.07, *SD* = 3.33, *n* = 36). The independent-samples *t*-test assuming equal variances showed a significant difference, *t*(70) = 5.10, *p* < 0.001, with a large effect size (Cohen’s *d* = 1.20).

Taken together, these results support H1 and suggest that, in this seminar context, collaborative homework was reliably associated with higher assignment performance than individual homework.

### 3.2 Task-specific collaborative differences (H2)

To test H2, we examined whether the performance advantage of collaboration varied across task types within the collaborative condition. Students in the collaborative condition performed significantly better on the concept-oriented assignment (*M* = 92.33, *SD* = 2.07, *n* = 18) than on the case-oriented assignments (*M* = 89.33, *SD* = 3.75, *n* = 36). Levene’s test indicated unequal variances, so Welch’s *t*-test was applied; the difference was statistically significant, *t*(51.35) = 3.79, *p* < 0.001. The effect size was moderate to large (Cohen’s *d* = 0.91), suggesting that collaboration was associated with greater performance gains on the concept-oriented task than on the case-oriented assignments. These results support H2 and indicate that the benefits of collaborative homework were more pronounced for the concept-oriented assignment, where tasks required higher levels of conceptual integration.

### 3.3 Perceived learning and qualitative interviews

To examine whether collaboration also shaped students’ perceived learning, independent-samples *t*-tests were conducted for each task type.

For the case-oriented tasks, collaborative work yielded higher ratings (*M* = 4.25, *SD* = 0.65, *n* = 36) than individual work (*M* = 3.81, *SD* = 1.06, *n* = 36). This difference was statistically significant, *t*(70) = 2.14, *p* = 0.036, with a small-to-moderate effect size (Cohen’s *d* = 0.50).

For the concept-oriented assignment, perceived learning ratings were also higher in the collaborative condition (*M* = 4.39, *SD* = 0.50, *n* = 18) than in the individual condition (*M* = 3.89, *SD* = 1.02, *n* = 18), *t*(34) = 1.86, *p* = 0.071, Cohen’s *d* = 0.62. Although this contrast did not reach conventional significance in this sample, the direction and magnitude of the effect were consistent with the performance results in favor of collaborative homework. For the case-oriented assignments, perceived learning was likewise analyzed at the episode level, so the same caveat regarding the approximate independence of episodes applies.

The qualitative data provided additional insight into how students experienced collaborative homework and helped explain the quantitative patterns. Thematic analysis yielded three interrelated themes, summarized in Table 3 along with representative translated excerpts. First, many students emphasized shared conceptual work and elaboration, describing how group discussion distributed cognitive load, prompted explanation of difficult concepts, and supported joint construction of more coherent understandings, especially for the concept-oriented assignment. Second, students highlighted perspective-taking and cognitive stimulation, noting that peers contributed different academic interests and interpretive angles that enriched case diagnoses and solution strategies and prompted them to reconsider initial assumptions. Third, students described both collaborative regulation and coordination costs: on the one hand, working together helped them structure the homework and clarify directions for action; on the other hand, they also reported challenges such as scheduling meetings, negotiating roles, and uneven participation. Even when such coordination costs were mentioned, students often concluded that the overall learning value of collaboration outweighed these difficulties.

**Table 3:** Themes identified from open-ended interview responses and representative quotes.

Theme	Description	Representative quotes
1. Shared conceptual work and elaboration	Students reported that peer explanations and joint discussion helped them clarify difficult concepts, integrate fragmented ideas, and construct more coherent understandings—particularly during the concept-oriented assignment	*“Listening to my groupmates expanded my thinking and deepened my understanding of the concept”; “The discussion helped me integrate scattered ideas into a more complete and well-organized answer”*
2. Perspective-taking and cognitive stimulation	Students highlighted exposure to diverse viewpoints and cognitive stimulation generated through group discussion. Hearing alternative interpretations prompted them to reconsider assumptions and develop new insights for both conceptual and case-oriented tasks	*“After hearing others’ interpretations, I could think about the problem from multiple angles instead of just my own”; “The conversation sparked insights that I would not have generated on my own”*
3. Collaborative regulation and coordination costs	Students described collaboration as offering scaffolding for starting, organizing, and completing the homework—especially for complex case-oriented tasks—but also noted challenges such as scheduling, role negotiation, and uneven participation. Overall, they perceived the learning benefits as outweighing these coordination costs	*“Our discussion provided a basic structure for the homework so that we knew how to proceed”; “Sometimes it was hard to find a time and balance everyone’s contributions, but in the end I still felt I learned more from working with the group”*

Themes were derived from students’ brief written open-ended interviews in the collaborative condition. Original responses were written in Chinese and were translated into English for reporting.

Together, these themes suggest that collaborative homework supported both cognitive and social processes—such as elaboration, perspective taking, and mutual regulation. These processes are consistent with Social Interdependence Theory and Socio-Cognitive Conflict Theory and help explain the observed advantages of collaboration in both performance and perceived learning.

## 4 Discussion and limitations

The present study examined how the design of post-discussion homework is related to learning in an authentic graduate seminar by jointly varying social architecture (collaborative vs. individual homework) and task type (concept-oriented vs. case-oriented). In this setting, collaborative homework was generally associated with better performance and perceived learning than individual homework, and this advantage was more pronounced for a concept-oriented assignment than for case-oriented assignments. By combining quantitative results with students’ brief written open-ended reflections on their collaborative homework experiences, the study illustrates how collaborative homework can carry forward inclusive, discussion-based learning beyond the classroom session.

Across all three assignments, students who completed homework collaboratively achieved higher performance than those who worked individually, supporting H1. This collaborative advantage was evident for both the concept-oriented assignment and the two case-oriented assignments, although the gap between collaborative and individual conditions was larger for the concept-oriented task. This pattern is consistent with work showing that cooperative structures tend to yield better academic outcomes than purely individual formats in higher education (Johnson and Johnson, 2002, 2009a; Kyndt et al., 2013; Slavin, 2014; Springer et al., 1999).

Analyses within the collaborative condition provided further support for H2. As outlined in the task design, the concept-oriented assignment was deliberately constructed as an ill-structured, high-integration task, whereas the case-oriented assignments primarily required applying established concepts to structured scenarios. When students worked in groups, performance on the concept-oriented assignment exceeded performance on the two collaborative case assignments by a moderate-to-large margin. In other words, collaboration was beneficial for both task types, but it was especially advantageous when students were required to reorganize and articulate theoretical ideas rather than apply familiar concepts to specific cases (Jonassen, 2000; Reed, 2016).

Perceived learning ratings broadly paralleled these performance patterns. Students reported that collaborative homework on the case-oriented tasks helped them understand and make sense of course content more than individual homework, and a similar—though statistically weaker—trend emerged for the concept-oriented assignment. Although the latter contrast did not reach conventional significance in this sample, its direction and effect size were consistent with the performance data. This convergence between objective performance and perceived learning echoes studies in cooperative learning that have found parallel gains in achievement and self-reported learning value (Ahmed Abdel-Al Ibrahim et al., 2023; Springer et al., 1999).

Taken together, these results extend the application of Social Interdependence Theory and Socio-Cognitive Conflict Theory to the domain of post-discussion homework (Butera et al., 2019; Deutsch, 1949; Johnson and Johnson, 2005, 2009a). From a social interdependence perspective, prior work has shown that positive interdependence and individual accountability can enhance learning in in-class group activities (Johnson and Johnson, 2002, 2009a). Structuring homework as a group product appears to activate similar dynamics beyond the classroom session: students in the collaborative condition described coordinating their efforts toward a shared outcome, monitoring one another’s contributions, and feeling jointly responsible for the quality of their work. This pattern is reflected in higher performance than in the individual condition across both task types.

The fact that collaborative advantages were more pronounced for the concept-oriented assignment highlights the importance of the fit between task demands and social architecture. In this study, collaboration was especially beneficial when homework required students to construct and reorganize conceptual understanding, rather than primarily apply a familiar framework to a specific case. This observation dovetails with work suggesting that collaborative structures are particularly effective when tasks call for high-level cognitive processing—such as integrating perspectives, comparing theoretical positions, or building conceptual frameworks—rather than simple reproduction of learned material (Richey et al., 2018; Scager et al., 2016; Stewart and Barrick, 2000). The findings thus complement prior research by showing, in an authentic graduate seminar, that both whether students collaborate and which tasks are assigned to collaboration matter for learning.

From the standpoint of Socio-Cognitive Conflict Theory, the combination of quantitative and qualitative evidence points to epistemic conflict and elaboration as key processes. The concept-oriented assignment was deliberately ill-structured and conceptually demanding, requiring students to integrate perspectives on “learning organization” and related theories. In their written open-ended interviews, students described how explaining concepts to peers, hearing alternative interpretations, and trying to reconcile divergent views prompted them to revisit initial assumptions and move from fragmented ideas toward more coherent explanations. Such accounts are consistent with the notion that exposure to discrepant viewpoints, followed by joint efforts to resolve them, can catalyze deeper conceptual restructuring (Butera et al., 2019; Johnson and Johnson, 2009b; Malone and Michael, 2018; Rodriguez-Dono and Hernández-Fernández, 2021). The stronger collaborative advantage for the concept-oriented assignment is in line with this theoretical perspective, insofar as conceptually open-ended tasks provide more room for socio-cognitive conflict and elaborative dialog to translate into improved understanding.

The qualitative themes also help clarify not only how collaborative homework supported learning but also what coordination costs accompanied these benefits. Students frequently described shared conceptual work and elaboration, as well as perspective-taking and cognitive stimulation, but they also mentioned practical challenges such as scheduling meetings, negotiating roles, and addressing uneven participation. These experiences echo prior work noting that group work can introduce coordination demands and concerns about equity and motivation (Barron, 2003; Radkowitsch et al., 2020; Yang, 2023). In this seminar, however, students typically framed these issues as manageable costs that were outweighed by the perceived learning benefits of collaboration, suggesting that a modest amount of well-structured collaborative homework can still yield substantial cognitive and social gains.

Practically, the findings offer a useful heuristic for designing post-discussion homework in graduate-level, discussion-based courses. Rather than treating homework as an isolated individual assessment, it can be designed as a continuation of the collaborative learning cycle. The advantages of collaborative homework observed here suggest that extending collaboration beyond the classroom can be beneficial when assignments are tightly connected to prior discussion. Group homework provided opportunities for students to revisit key ideas, elaborate on classroom dialog, and jointly construct written products that consolidated their understanding (Cohen, 1994; Premo et al., 2018; Springer et al., 1999). At the same time, the pattern of results underscores that collaborative work should be aligned with the learning goal. When the aim is to foster high-level conceptual thinking—such as constructing frameworks, comparing theoretical perspectives, or interrogating core constructs—collaborative homework appears particularly effective. For case-oriented tasks, collaboration also generated performance and perceived learning gains, but the qualitative findings suggest that these tasks may require more explicit attention to group processes to prevent coordination problems from undermining motivation. In this way, carefully designed collaborative homework can serve as one concrete tool for supporting cognitive and social inclusion beyond classroom discussion.

Several limitations of this study should be borne in mind when interpreting the findings. First, the sample consisted of 36 psychology graduate students from a single seminar at a public comprehensive university in China. This small and relatively homogeneous sample constrains statistical power and limits generalizability. The results are therefore best viewed as indicative of patterns in similar graduate-level, discussion-based courses rather than as definitive estimates for broader populations or disciplines.

Second, the study employed a quasi-experimental design within an authentic course and relied on intact project teams. Although teams were randomly assigned to counterbalanced sequences and the order of collaborative versus individual homework was controlled at the team level, students worked in stable teams throughout the semester and no baseline achievement or additional covariates (e.g., prior GPA, prior collaboration experience) were collected. As a result, the observed differences between collaborative and individual homework are most appropriately interpreted as associations observed under these instructional conditions, rather than as strictly causal effects.

Third, our analytic strategy balanced ecological validity and statistical refinement. To reflect the way homework is experienced across the semester and to retain sufficient statistical power in this modest sample, we treated assignment submissions as performance episodes and conducted episode-level comparisons, complemented by aggregated student-level checks. This choice preserves the temporal and curricular structure of the tasks, but it does not fully model the nesting of episodes within students and students within groups. Future research with larger samples could extend this work by applying multilevel or repeated-measures models to more explicitly capture the hierarchical structure and potentially provide more conservative estimates.

Fourth, the measurement strategy focused on rubric-based performance scores and a brief indicator of perceived learning. Double marking with acceptable inter-rater reliability strengthens confidence in the performance scores, but they remain situated within one course and one instructor. The single-item perceived-learning measure was intentionally concise to minimize disruption in an authentic teaching context, yet it cannot capture the full multidimensionality of students’ learning experiences. In addition, in collaborative conditions a shared group score was applied to all team members, which reduces within-group variance and may lead to somewhat larger standardized effect size estimates. Future studies could incorporate multi-item scales (e.g., for perceived learning, cognitive load, and self-efficacy) and adopt designs that allow for separate individual and group-level scoring.

Finally, task type in this study was implemented as part of the course design and treated analytically as a proxy for cognitive demand rather than as a directly measured construct. The distinction between concept-oriented and case-oriented assignments is grounded in work on ill-structured versus well-structured problems, but we did not collect direct measures of perceived task difficulty or cognitive load. Caution is therefore warranted when interpreting the task-specific collaborative differences as reflecting cognitive demand per se. Future research could incorporate short cognitive load scales or manipulate task features more systematically to test more precisely when collaborative advantages are amplified. In addition, the qualitative component relied on brief written reflections collected after collaborative assignments; these accounts offer valuable insight into students’ perceptions but do not provide fine-grained process data on how socio-cognitive conflict and regulation unfolded during homework. Subsequent studies could complement written reflections with recordings, collaboration logs, or discourse analyses to trace these processes in greater detail.

Despite these limitations, the study reframes post-discussion homework not as an isolated individual add-on but as a dynamic phase of the collaborative learning cycle. By intentionally designing assignments that take into account both social architecture and cognitive demand, educators can extend the benefits of inclusive, discussion-based pedagogy beyond the classroom. The critical question for practice thus shifts from whether students should work together after class to a more thoughtful consideration of how they should collaborate and on what kinds of tasks.

## 5 Conclusion

Taken together, the findings indicate that in an authentic graduate seminar, the learning value of post-discussion homework depends on how social architecture and task type are combined. Across assignments, collaborative homework was associated with better outcomes than individual homework, and this advantage was especially pronounced when students engaged in concept-oriented tasks that required them to reorganize and articulate theoretical ideas. Students’ perceived learning ratings and qualitative interviews converged with this pattern. Conceptually, the findings extend Social Interdependence Theory and Socio-Cognitive Conflict Theory into the homework phase, suggesting that collaborative homework is most powerful when it sustains shared responsibility and creates space for conceptual integration and productive disagreement. In this sense, collaborative homework can serve as a modest but practical tool for supporting cognitive and social inclusion beyond class meetings. Practically, these findings point to a clear design implication: treat post-discussion homework as a continuation of classroom dialog, assign the more conceptually demanding tasks to collaborative formats, and provide basic support for group coordination. Within the constraints of a small quasi-experimental study in a single graduate course, these conclusions are necessarily bounded, but they highlight homework as a deliberate phase of the collaborative learning cycle rather than a routine individual add-on in otherwise discussion-based courses.

## Data Availability

The original contributions presented in the study are included in the article/supplementary material, further inquiries can be directed to the corresponding author.
